# Stranger in a strange land: the experiences of immigrant researchers

**DOI:** 10.1186/s13059-017-1370-4

**Published:** 2017-12-15

**Authors:** Sophien Kamoun, Rosa Lozano-Durán, Luay Nakhleh

**Affiliations:** 1 The Sainsbury Laboratory, Norwich Research Park, Norwich, NR4 7UH UK; 2 Shanghai Center for Plant Stress Biology, Chinese Academy of Sciences, Shanghai, 201602 China; 3 Department of Computer Science, Rice University, 6100 Main Street, Houston, TX 77005 USA

## Abstract

Continuing with our Q&A series discussing issues of diversity in STEM fields, *Genome Biology* spoke with three researchers on their experiences as immigrants.

International collaborations are key to advancing scientific research globally and often require mobility on the part of researchers. Migration of scientists enables the spread of ideas and skills around the world, giving researchers the opportunity to follow the best resources. Of course, migration adds a new set of challenges to the already monumental task of starting and running a lab. *Genome Biology* spoke to Sophien Kamoun, Rosa Lozano-Durán, and Luay Nakhleh about their personal experiences.

## What influenced your choice to move to your current country?

**SK**: There is this old German expression “wo die Musik spielt”—you go where it’s happening, where the “music is played”. I think that sums it up. When I was a student in the 1980s, almost everyone wanted to do a Ph.D. in the USA. I felt that to have the best training and to be among the best, I had no choice but to study in the USA. I think that was a pretty correct assessment of the state of affairs in the 1980s. Indeed, I had a fantastic experience at the University of California, Davis. Also, at that time, Europe wasn’t really open to non-Western scientists, and international mobility wasn’t recognized like it is today [1]. Later, I moved to the Netherlands and then back to the USA before landing in my current position at The Sainsbury Laboratory (TSL) in Norwich, UK. I moved to Norwich exactly 10 years ago, primarily because of the reputation of the laboratory as a center of excellence for plant pathology research and the generous support provided by David Sainsbury through the Gatsby Foundation. I have had a phenomenal time at TSL these past 10 years, where I have had the opportunity to work with outstanding scientists from perhaps about 30–40 countries. An interesting point is that when TSL was founded in 1988, all the group leaders were British [2], but currently our principal investigators are from all over the world [3]. I think TSL truly reflects the emergence of the #ScienceisGlobal movement on social media [4], which is so evident in the UK and other corners of Europe.

**RL-D**: Three years ago, having worked as a postdoctoral researcher for almost four years, I was eager to establish my own laboratory. I had known what I wanted to devote my research to for a long time and could not wait to get started. Unfortunately, the economic climate in Europe, where I am originally from and where I was working at the time, was not particularly propitious for science in academia, with research budgets being slashed and increasing competition—not the most favorable situation for new group leaders, I heard over and over again. My partner was also a scientist at the same career stage, and so we needed to find two positions, not just one, complicating matters even more. One day, just by chance, we came across a job advertisement for group leader positions at the Shanghai Center for Plant Stress Biology in China. We had heard about the place—a new institute with the ambition to become a powerhouse for plant sciences. I was very excited at the prospects of leading my own research group, and that excitement overrode any qualms or self-imposed geographical restrictions. I am also fortunate enough to have an incredibly supportive family and friends who unconditionally encouraged me to pursue my scientific career, even if that involved moving far away; they may not always understand the nitty-gritty details of what I do, but they know how important it is for me.

It was my first job application, and I was offered the position following an interview at the center. They were willing to support me and give me the freedom to develop my own research program—it was an unbeatable opportunity to start my independent career. And the fact that I would be living in Asia, with the immense chance to broaden my experience that entailed, added some extra appeal (despite the slight vertigo I also felt). There was not much to think about, really—it was a deal I simply could not turn down.

**LN**: I was born to a Christian Arab family in Israel and did my undergraduate studies at the Technion (Israel Institute of Technology). Although I was an atheist by the time I started my studies at the Technion, I still considered myself to be “culturally” Christian, in that I celebrated Christmas and New Year with my family (eating and drinking, not going to church!). However, almost every year, my exams were scheduled on December 25^th^ and January 1^st^ (the Fall semester in Israel starts in October and ends in February). Being unable to take exams on different dates affected my performance in my studies and my interest in pursuing graduate studies at the same institution. Also, more generally, I was the only Christian Arab student in my class, and one of a handful of Arab students; I never felt comfortable at the time. So, I decided to pursue graduate studies in computer science outside Israel. The choice to come to the USA was an easy one because the USA had (and still has, in my opinion) the best graduate programs in computer science.

## Can you comment on any barriers or unconscious biases you have experienced as an immigrant scientist that perhaps your colleagues have not experienced?

**SK**: It is difficult to make such claims with confidence and without sounding like a complainer or a grouch. There is a fine line between paranoia and perception. Perhaps this question is best asked to our native colleagues and whether they feel they are biased. This said, we all have cognitive biases, particularly feelings outside of conscious awareness [5]. Professor Uta Frith has developed an outstanding animation and briefing on unconscious bias that I urge everyone to consult [6]. The best we can do is to educate and create processes to remind colleagues, committees, funders, etc. that we shouldn’t judge people on the basis of ethnicity, gender, physical appearance, disability, or other things people have no control over. What I find most useful is to raise awareness about valuing diversity in all its forms and to think of actions and incentives for improving behavior.

**RL-D**: The language and cultural barriers can be pervasive and easily penetrate the work environment. When you move to a new country, you will inevitably make a lot of wrong assumptions regarding how things work—you will soon realize ways are less universal than you presumed and might ultimately be reduced back to the most basic trial-and-error approach. This can reach every aspect of your work life. Applying for national funding might be an ordeal when you can only get the second-hand information that was not lost in translation, and undergraduates may become extremely confused when you don’t treat them according to the accepted unwritten code of conduct for supervisor–student relations. If you don’t speak the language, this will certainly limit your interactions with colleagues, pruning scientific discussion. Sometimes people (scientists included) don’t feel too comfortable speaking English when it’s not their mother tongue, and they will simply avoid it. Of course, learning the language is part of the effort that trying to integrate into a new country requires, but it can be challenging to find the time and energy to do so when you are establishing your new research group. The sensation that you are missing essential information to aptly navigate the system, with the concomitant degree of disorientation, is ever-present—it ends up being the backdrop of your working days. Luckily, you will find people around you willing to go the extra mile to help. I have been privileged to have received the support and encouragement of some incredibly helpful staff members at the institute, fantastic colleagues, an amazing laboratory manager, and a wonderful group of students and postdocs that make the most vibrant, energetic team.

On the other hand, there are also specific advantages to being a foreign scientist. Because you are different, you are more likely to be remembered, so getting your own identity in the scientific community might happen sooner than somewhere where you tend to blend into the background—you might be associated to your research topic more easily and effortlessly. Also, the fact that you obtained your scientific education abroad means you often have a different perspective or a complementary set of skills, which is usually highly valued and favors fruitful cooperation with other researchers.

**LN**: I cannot think of any. In fact, a large number (if not the majority) of graduate students in computer science departments are international students. Many faculty members in computer science departments are immigrant scientists. This is why I felt very comfortable studying in the USA; I have been surrounded by students and scientists with backgrounds similar to mine.

## What have been your biggest challenges and greatest opportunities in your career?

**SK**: In most countries, the bar tends to be higher if you are an immigrant. You can’t be just as good as the next candidate to get the job or the promotion. However, this can also be a great motivator to be better and excel. You just have to believe that you live in a meritocracy and that the better candidate will be recognized. Personally, I generally felt that being an immigrant has enabled me to forge my own style and personality without worrying too much about other people’s expectations. Being an immigrant actually comes with some degree of freedom. Given that people expect you to be different, you may as well be yourself.

**RL-D**: As is often the case, the answer to both questions would be the same: moving to China has been both the greatest challenge and the greatest opportunity. The move has been, without a doubt, the greatest opportunity in my career so far. Here, I’ve had the chance to set up my own laboratory and start my independent research project in unbeatable conditions. I work with a fantastic team and great collaborators, and we have the means and the freedom to develop our research lines and explore new directions together—seeing our projects unfold is incredibly exciting. I had never enjoyed doing science as much as I do now every single day.

As it is easy to imagine, though, it came with a toll to pay. Changing country necessarily meant I was pushing myself out of my comfort zone, with all the fears and uncertainties that entails. I had lived away from my native Spain before, in the USA and in the UK, but moving to China was a bigger challenge, simply because the cultural differences are obviously larger. And no matter how much you try to prepare yourself, how much you read and ask around and think about the future, the truth is you never really know (and therefore are never prepared for) what is coming your way. Being suddenly immersed in an unfamiliar environment can feel alienating and lonely. I suddenly discovered I needed help navigating the simplest aspects of life, which made me feel terribly dependent. Unexpectedly, daily life outside the lab became my main source of stress!

Naturally, this was just the initial acclimation stage, and as such it was temporary and eventually faded away. The lab was growing and developing, I had a group of friends in Shanghai who are my family away from my family, and it soon became evident to me that the advantages far outweigh the troubles. I think the greatest opportunities frequently present themselves wrapped in the biggest challenges; they cannot be reached until you go through the layers of difficulties and risks.

**LN**: The Arab culture, even for Arabs in Israel, is very different from that of the USA. The biggest challenge was adjusting to the culture in the USA, including at work. The greatest opportunities for me have been to complete my graduate studies in premiere academic institutions, join the faculty of another premiere institution (Rice University, USA), and rise through the academic ranks without ever feeling any barriers or biases against me. I have always felt that whatever I was capable of achieving and willing to work towards was accessible to me in the same way it was accessible to any of my colleagues.

## What more can be done to encourage and support researchers living overseas?

**SK**: There is much effort to raise awareness about gender balance and related issues in science. This is of course highly commendable, and it is hopefully resulting in concrete outcomes to improve the roles that women play in science and academia. I wish to see a similar movement towards appreciating the impact of foreign-born scientists. We need to better recognize that ethnic and cultural diversity brings a lot of positives to the scientific enterprise. For one thing, it is well established that multicultural experiences tend to increase creativity and are more likely to generate that “aha” moment [7]. It also helps to counteract the infamous old-boy network that has plagued science and academia for ages [8].

I propose that the percentage of foreign-born scientists should be considered as a measure of diversity that is applied to committees and other decision bodies. There is a lot of trumpeting of #ScienceisGlobal by funding agencies and learned societies, but I’m not so sure that their diversity record in terms of foreign-born scientists is that great. An example would be the 2014/2015 rounds of the Biotechnology and Biological Sciences Research Council (BBSRC) Future Leader Fellowships, which has a great record for gender balance but the diversity of the selected candidates doesn’t seem to match the diversity of students and postdocs that I see around me [9]. I’m glad that the 2016 cohort seems to be more diverse. One step forward is to pay close attention to the composition of the selection committees. Does the committee diversity in terms of foreign-born members reflect the diversity of the institution? Another action is to educate committees about unconscious bias and how this may affect their decision making [6].

**RL-D**: When considering moving abroad, there are two barriers: psychological and practical. In my opinion, both of them can be made easier to overcome. Information is helpful in addressing the former: it is useful to hear about/from researchers who are or have been in a similar situation, and to learn about the difficulties they encountered and how they handled them. In this sense, promoting networking among foreign researchers can have a very positive impact, as well as publicizing examples of successful stories in an open and honest way. However, ensuring that there are people available to offer practical help and support where needed (from going to the doctor to writing a grant proposal) can not only improve the living and working conditions of immigrant scientists, but also provide a peace of mind that is essential to make the decision to move in the first place, and to stay mentally healthy in the new country afterwards. Facilitating integration would also be an important goal to be met. Institutions could, for example, provide language lessons and cultural orientation for foreign staff members. Other needs are not necessarily restricted to immigrant scientists, but can become particularly pressing when abroad. For example, scientists moving with partners and/or children will require additional flexibility and facilities, such as those offered by dual career schemes or the availability of childcare at the workplace.

The reason for supporting and promoting international experience among researchers is obvious: science benefits from diversity. Scientists from varied backgrounds, with different scientific upbringing and education, may offer complementary perspectives on scientific problems. Movement of scientists between countries not only impacts their individual careers, but on the larger scale also contributes to making science multicultural and to enable its growth through the integration of diverse approaches and ways of thinking.

**LN**: To me, the greatest aspect of working in academia in the West is the academic freedom that scholars are afforded to conduct their research and educate their students. In the USA and Western countries, we take this freedom for granted. Many researchers living overseas do not have the same freedom, and supporting them in this aspect is crucial. For example, I like to point to the excellent blog by Professor Rasmus Nielsen about support for teaching evolution in the Middle East [10], which also highlights some of the challenges in getting approval for grant proposals on the subject. Furthermore, researchers in many countries have the intellectual capacity to make significant contributions to science, yet they lack the funding or support. Exchange programs with American institutions benefit them tremendously. Facilitating, rather than restricting, visits by these scientists to the USA would be great support for them.

## Box 1. Contributors’ research interests

**Sophien Kamoun (SK):** I study how microbes and plants interact, how some microbes cause disease in plants, and how plants fight them off. I work primarily on blight and blast diseases of crops such as potato, rice, and wheat. I’m originally from Tunisia, and I currently live in the UK.

**Rosa Lozano-Durán (RL-D):** In my laboratory, we investigate how plant viruses cause disease. Viruses are fascinating: with an extremely limited toolbox, they have evolved to efficiently co-opt the cell’s molecular machinery for their own functions. We study how this viral manipulation occurs at the molecular level, how it ultimately results in an infection, and the strategies plants deploy to defend themselves. I am from Spain, and I currently live in China.

**Luay Nakhleh (LN):** My research falls in the general area of computational biology. My focus is on developing mathematical models and algorithmic techniques for analyzing biological, mainly genomic, data. In particular, I focus on questions on evolutionary biology. I am from Israel and currently living in the USA.
